# Evaluation of Antiviral Activity of Gemcitabine Derivatives against Influenza Virus and Severe Acute Respiratory Syndrome Coronavirus 2

**DOI:** 10.1021/acsinfecdis.3c00034

**Published:** 2023-03-13

**Authors:** Hyeon-Min Cha, Uk-Il Kim, Soo Bin Ahn, Myoung Kyu Lee, Haemi Lee, Hyungtae Bang, Yejin Jang, Seong Soon Kim, Myung Ae Bae, Kyungjin Kim, Meehyein Kim

**Affiliations:** † Infectious Diseases Therapeutic Research Center, Korea Research Institute of Chemical Technology (KRICT), Daejeon 34114, Republic of Korea; ‡ Graduate School of New Drug Discovery and Development, Chungnam National University, Daejeon 34134, Republic of Korea; § ST Pharm Co., Ltd., Seoul 06194, Republic of Korea; ∥ College of Pharmacy, Dongguk University, Goyang-si, Gyeonggi-do 10326, Republic of Korea; ⊥ Drug Discovery Platform Research Center, Korea Research Institute of Chemical Technology (KRICT), Daejeon 34114, Republic of Korea

**Keywords:** influenza virus, SARS-CoV-2, antiviral agent, polymerase inhibitor, gemcitabine derivatives

## Abstract

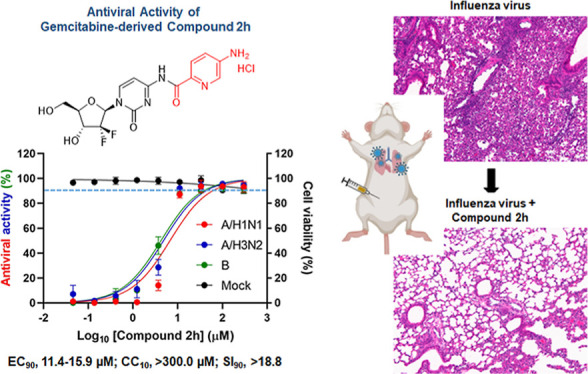

Gemcitabine is a nucleoside analogue of deoxycytidine and has been reported to be a broad-spectrum antiviral agent against both DNA and RNA viruses. Screening of a nucleos(t)ide analogue-focused library identified gemcitabine and its derivatives (compounds **1**, **2a**, and **3a**) blocking influenza virus infection. To improve their antiviral selectivity by reducing cytotoxicity, 14 additional derivatives were synthesized in which the pyridine rings of **2a** and **3a** were chemically modified. Structure-and-activity and structure-and-toxicity relationship studies demonstrated that compounds **2e** and **2h** were most potent against influenza A and B viruses but minimally cytotoxic. It is noteworthy that in contrast to cytotoxic gemcitabine, they inhibited viral infection with 90% effective concentrations of 14.5–34.3 and 11.4–15.9 μM, respectively, maintaining viability of mock-infected cells over 90% at 300 μM. Resulting antiviral selectivity was comparable to that of a clinically approved nucleoside analogue, favipiravir. The cell-based viral polymerase assay proved the mode-of-action of **2e** and **2h** targeting viral RNA replication and/or transcription. In a murine influenza A virus-infection model, intraperitoneal administration of **2h** not only reduced viral RNA level in the lungs but also alleviated infection-mediated pulmonary infiltrates. In addition, it inhibited replication of severe acute respiratory syndrome virus 2 infection in human lung cells at subtoxic concentrations. The present study could provide a medicinal chemistry framework for the synthesis of a new class of viral polymerase inhibitors.

Influenza virus, a major cause of respiratory disease in humans, belongs to the family *Orthomyxoviridae* and has eight segmented, negative-sense RNA genomes.^1^ To invade cells, viral hemagglutinin recognizes cell surface receptors, α2,3- and α2,6-typed sialic acids, promoting receptor-mediated endocytosis.^2^ Viral ribonucleoprotein (vRNP) complexes, composed of viral RNA, nucleoprotein (NP), polymerase basic protein 2 (PB2), PB1, and polymerase acidic protein (PA), are subsequently released to migrate into the nucleus where robust genome replication occurs.^3,4^ As another respiratory pathogen, severe acute respiratory syndrome virus 2 (SARS-CoV-2), belonging to the group of *Betacoronaviruses*, has been identified as the causative agent of coronavirus disease 2019 (COVID-19).^5^ Within the viral particle, single-stranded positive-sense RNA genome of about 30 kb is complexed with nucleocapsid protein, forming vRNP complex.^6^ Intracellular entry of SARS-CoV-2 is triggered by binding of the receptor-binding domain within the spike protein (S) to the cell surface receptor named angiotensin-converting enzyme 2.^7^ After virus internalization, the viral genomic RNA (gRNA) with a 5′-cap structure and a 3′-poly(A) tail primarily translates the two polyproteins, pp1a and pp1ab. Among the 16 mature nonstructural proteins (nsps) generated by proteolytic cleavage of the polyproteins, nsp7, nsp8, and nsp12 compose viral RNA polymerase complex.^8,9^ When viral gRNA binds to the polymerase complex, the RdRp domain of nsp12 facilitates synthesis of negative-sense genomic and subgenomic RNA intermediates, which serve as templates for transcription of positive-sense gRNA and at least nine subgenomic mRNAs.^10,11^

After virus enters host cells, RNA-dependent RNA synthesis is one of the most crucial steps for viral genome amplification or for gene expression and thus has been regarded as a primary target for antiviral development irrespective of genome polarity. In this context, it is inevitable that strategies of antiviral drug discovery place high priority on synthesis of nucleos(t)ide analogues. To date, there are two classes of approved antivirals inhibiting influenza viral genome amplification or transcription, including baloxavir marboxil and favipiravir (also named T-705). Baloxovir marboxil, a non-nucleoside analogue, targets the endonuclease activity of PA, while favipiravir, a purine nucleoside analogue, blocks RNA-dependent RNA polymerase, PB1, after converting into the ribofuranosyl 5′ triphosphate-active form.^12,13^ During COVID-19 pandemic, remdesivir (RDV) and molnupiravir (also named MK-4482 or EIDD-2801) that are adenosine and cytidine analogues, respectively, have been approved for emergency use against SARS-CoV-2.^14,15^ In contrast to RDV, molnupiravir has received conditional marketing authorization in the United Kingdom. It is because of the fact that molnupiravir not only can be incorporated into viral RNAs during viral genome replication, resulting in lethal mutagenesis of SARS-CoV-2, but also can lead to host DNA mutations undesirably.^16^ Nevertheless, diversifying antiviral portfolio by developing alternative broad-spectrum, nucleos(t)ide analogues has been suggested to be the most effective way to pandemic preparedness against newly emerging RNA viruses.

For the treatment of influenza virus or SARS-CoV-2 infections, besides favipiravir, RDV, and molnupiravir, other nucleos(t)ide analogues have been designed and investigated. Representatively, gemcitabine, a cytidine analogue with a chemical structure of 2′,2′-difluoro-2′-deoxycytidine, has been reported to inhibit infection of different kinds of DNA or RNA viruses.^17−20^ Originally, it was developed for treatment of solid tumors by arresting growth of carcinoma cells.^21^ Pharmacokinetic studies demonstrated that gemcitabine and its deaminated intracellular metabolite, 2′,2′-difluoro-2′-deoxyuridine (dFdU), can be converted into their cognate triphosphates to be incorporated into host DNA as well as RNA.^22^ Given these pharmacological properties, it is not surprising that even though gemcitabine is antivirally active, there is a concern about transient or sometimes irreversible cytotoxicity. It can be exemplified by a clinical study in which oral administration of gemcitabine resulted in liver necrosis-mediated lethal hepatotoxicity.^23^ These findings stress the importance of chemical modification of gemcitabine to increase antiviral selectivity by reducing cytotoxicity.

On the basis of chemical structures of primary hits from screening of an in-house nucleos(t)ide analogue-focused library, we synthesized 14 compounds by modification of the amine group of gemcitabine. The aim of our study was to optimize gemcitabine to have reduced cytotoxicity but antiviral effectiveness comparable to a clinically available nucleoside analogue. To ensure improved safety, we assessed antiviral selectivity indices by measuring the 90% effective concentrations (EC_90_) as well as 10% cytotoxic concentrations (CC_10_). This high-standard evaluation enabled identification of a more-potent, less-toxic cytidine derivative, **2h**. It could provide a framework for the discovery of a new class of safer nucleoside analogues inhibiting both negative- and positive-sense RNA viruses.

## Results

### Identification of Gemcitabine Derivatives as Anti-Influenza Viral Agents from a Nucleos(t)ide Analogue-Focused Chemical Library

**Figure 1:**
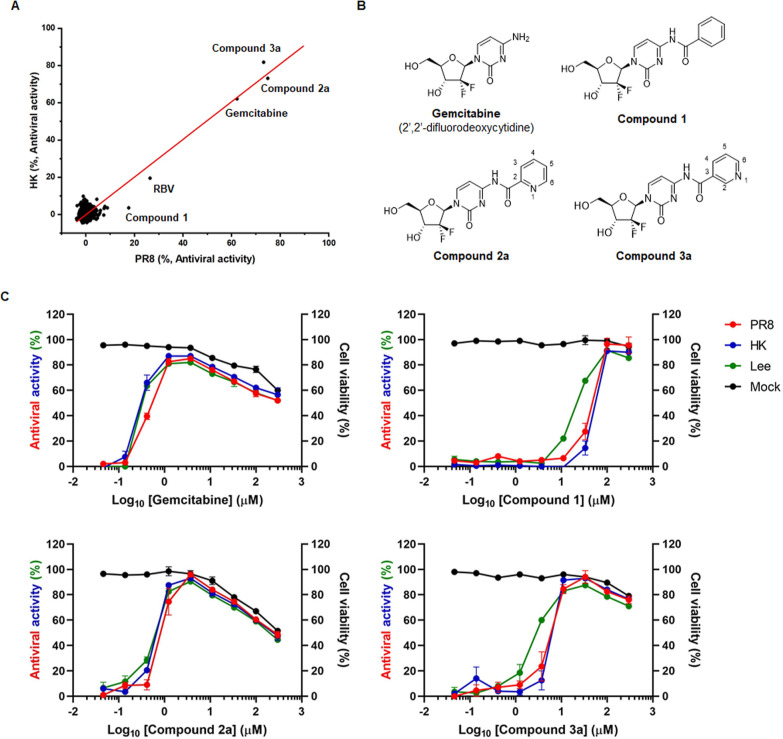
Screening of a chemical library for identification of hit compounds against influenza virus. (A) Cell-based antiviral assay against influenza A viruses. MDCK cells infected with influenza A virus, either A/Puerto Rico/8/34 (H1N1; PR8) or A/Hong Kong/8/68 (H3N2; HK), at a multiplicity of infection (MOI) of 0.001, were treated with 10 μM of each compound from a chemical library composed of 488 nucleoside analogues. On day 3 postinfection, cell viability was measured using 3-(4, 5-dimethylthiazolyl-2)-2, 5-diphenyltetrazolium bromide (MTT). Values from mock-infected and virus-infected, mock-treated cells were defined as 100 and 0%, respectively. Ten micromolar concentration of RBV was used as an internal control. The scatter plot shows relative antiviral activity (%) of each compound against the two viruses. Active compounds of interest are labeled. (B) Chemical structure of gemcitabine and its derivatives, **1**, **2a**, and **3a**, identified from the cell culture-based screening against influenza A viruses, as shown in (A). (C) Dose–response curve of antiviral activity and cytotoxicity of gemcitabine and the hit compounds. MDCK cells were mock-infected (black) or infected with three viruses, PR8 (red), HK (blue), and B/Lee/40 (Lee; green), individually at an MOI of 0.001 for 1 h at 35 °C. After removal of unabsorbed virus, they were treated with threefold serial dilutions (from 300 to 0.05 μM) of each compound among gemcitabine (upper left), **1** (upper right), **2a** (lower left), and **3a** (lower right). On day 3, relative antiviral activity (left *y*-axis) and cell viability (right *y*-axis) were determined using an MTT assay in which values from 0.6% DMSO-treated cells and 0.6% DMSO-treated, virus-infected cells were defined 100 and 0%, respectively. Values are expressed as means ± SEM from three different samples.

To discover compounds active against influenza A viruses, 10 μM of each compound from a chemical library composed of 488 nucleos(t)ide derivatives was treated to A/Puerto Rico/8/34 (PR8; A/H1N1) or A/Hong Kong/8/68 (HK; H3N2) virus-infected Madin–Darby canine kidney (MDCK) cells. Dimethyl sulfoxide (DMSO) (0.2%) and 10 μM ribavirin (RBV) that inhibits viral polymerase activity were used as negative and positive controls, respectively. Three days after compound treatment, cell viability was measured from mock- or virus-infected cells to measure inhibitory effect (Figure 1A). Screening results demonstrated that among the test compounds, gemcitabine, **2a** and **3a** improved viability of both PR8- and HK-infected cells to over 50%, whereas compound **1** had a marginal effect, with 10–20% remaining viable cells at the concentration tested. Structural analysis demonstrated that compounds **1**, **2a**, and **3a** are gemcitabine derivatives with benzoyl, picolinoyl, and nicotinoyl moieties, respectively, at the amine group (Figure 1B).

We analyzed their dose–response curves to quantitatively determine antiviral efficacy and cytotoxicity after synthesizing the hit compounds of high purity (>95%). Increasing concentrations from 0.05 to 300 μM were treated to MDCK cells mock-infected or infected with influenza A viruses, PR8 and HK, or influenza B virus, the B/Lee/40 strain (Lee) (Figure 1C and Tables 1 and 2). In the cytopathic effect (CPE) experiment, four known compounds with various modes of antiviral action were used as controls, including RBV and favipiravir, which target PB1; amantadine (AMT), which inhibits the viral M2 ion channel; and oseltamivir carboxylate (OSV-C), which blocks NA activity. Selectivity index (SI_50_) values indicated that gemcitabine more selectively inhibited influenza viruses (50% effective concentration [EC_50_] values ranging between 0.3 and 0.7 μM, 50% cytotoxic concentration [CC_50_] value above 300 μM, and resulting SI_50_ values above 464.8) than the hit compounds, **1** (EC_50_, 22.5–54.2 μM; CC_50_, >300.0 μM; and SI_50_, >5.5) and **3a** (EC_50_, 3.1–6.2 μM; CC_50_, >300.0 μM; and SI_50_, >48.4) (Tables 1 and 2). It was comparable to **2a** (EC_50_, 0.6–0.9 μM; CC_50_, >300.0 μM; and SI_50_, >342.5). It was noteworthy that in contrast to gemcitabine which failed to reach cell viability above 90% after influenza virus infection, compounds **1** and **2a** successfully recorded over 90% viability of infected cells at subtoxic concentrations, 100 and 3.7 μM, respectively (Figure 1C). Attributing to the improved cell viability, higher-standard antiviral selectivity indices were applicable for compounds **1** (90% effective concentration [EC_90_], 76.2–98.1 μM; 10% cytotoxic concentration [CC_10_], >300.0 μM; and SI_90_, >1.9) and **2a** (EC_90_, 1.4–2.2 μM; CC_10_, 9.9 μM; and SI_90_, 4.4–7.1) (Table 1). With the same criteria, compound **3a** was active against influenza A viruses (EC_90_, 10.6–16.9 μM; CC_10_, 44.4 μM; and SI_90_, 2.7–4.2) but not against influenza B virus (SI_90_, not detected). Nevertheless, together with compounds **1** and **2a**, compound **3a** was included in candidate compounds for further modification because of its ninefold reduced cytotoxicity as mentioned (CC_10_ values: 5.0 μM for gemcitabine versus 44.4 μM for **3a**) (Table 2). These findings indicated that substitution of the amine group of gemcitabine with an aromatic carbonyl group would be advantageous to improve antiviral selectivity.

**Table 1:**
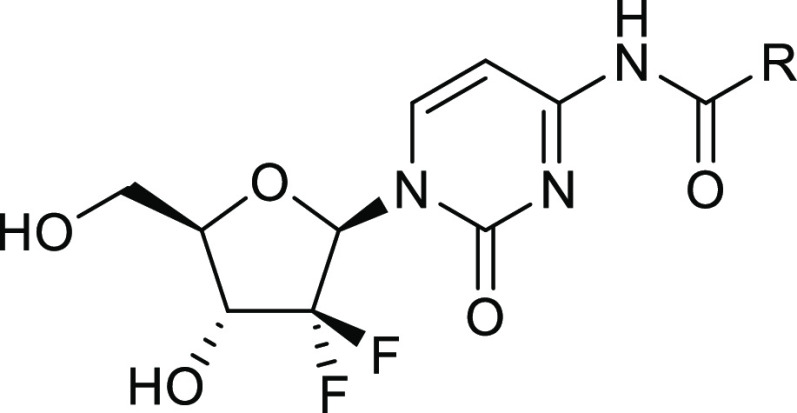

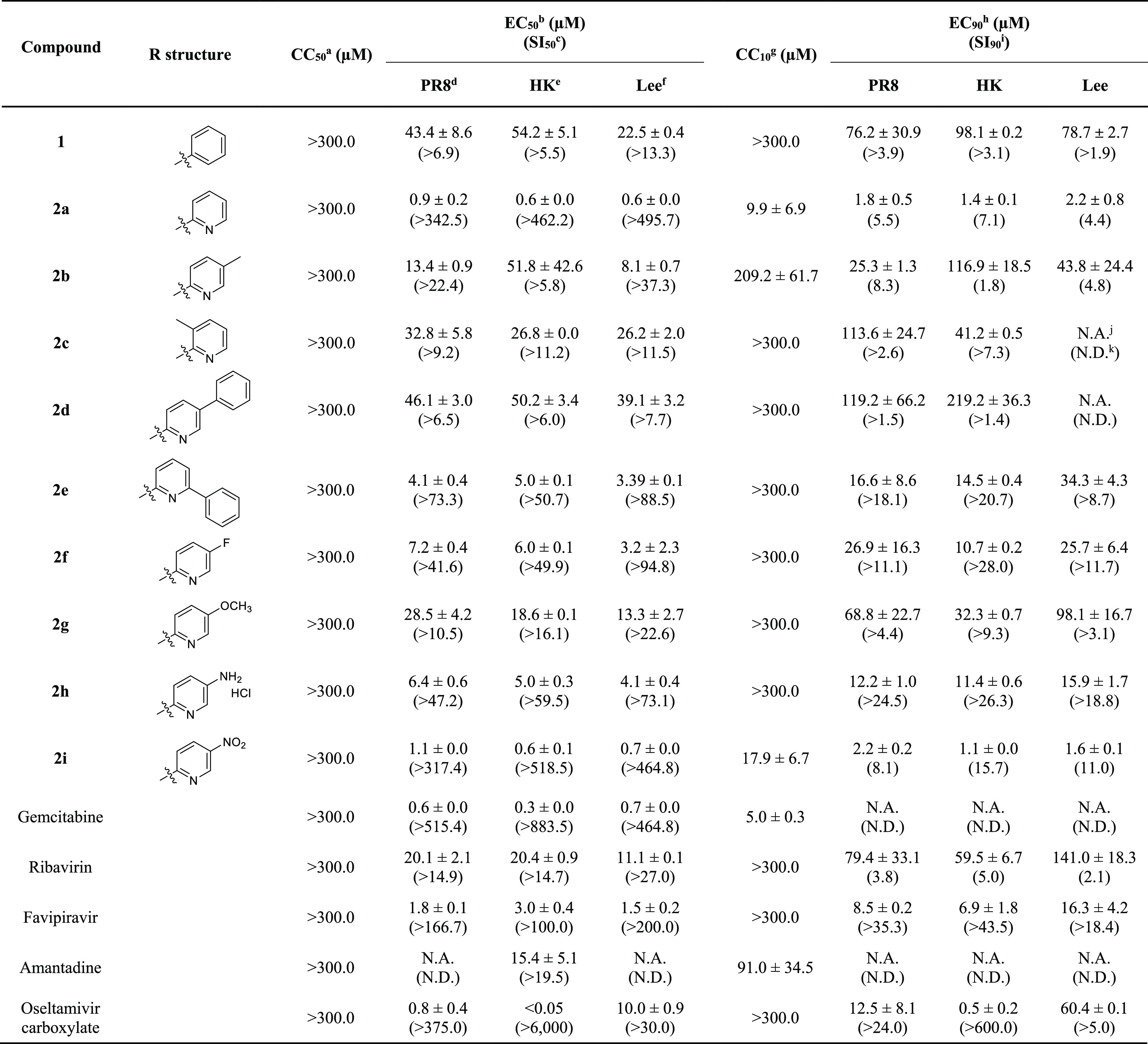
Antiviral Activity of Pyridine-2-carbonyl Substituents

a Compound concentration required for inhibiting cell viability by 50%.

b Compound concentration required for enhancing viability of each virus-infected cells to 50%.

c The ratio of CC_50_ to EC_50_.

d A/Puerto Rico/8/34 (H1N1).

e A/Hong Kong/8/68 (H3N2).

f B/Lee/40.

g Compound concentration required for inhibiting cell viability by 10%.

h Compound concentration required for enhancing viability of each virus-infected cells to 90%.

i The ratio of CC_10_ to EC_90_.

j Not available.

k Not determined.

**Table 2:**
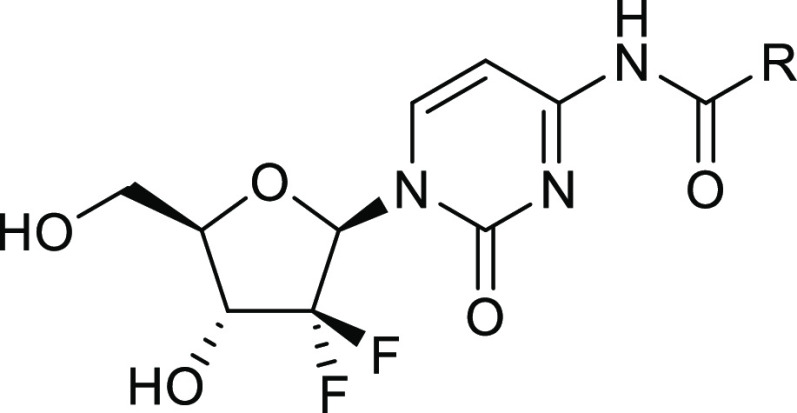

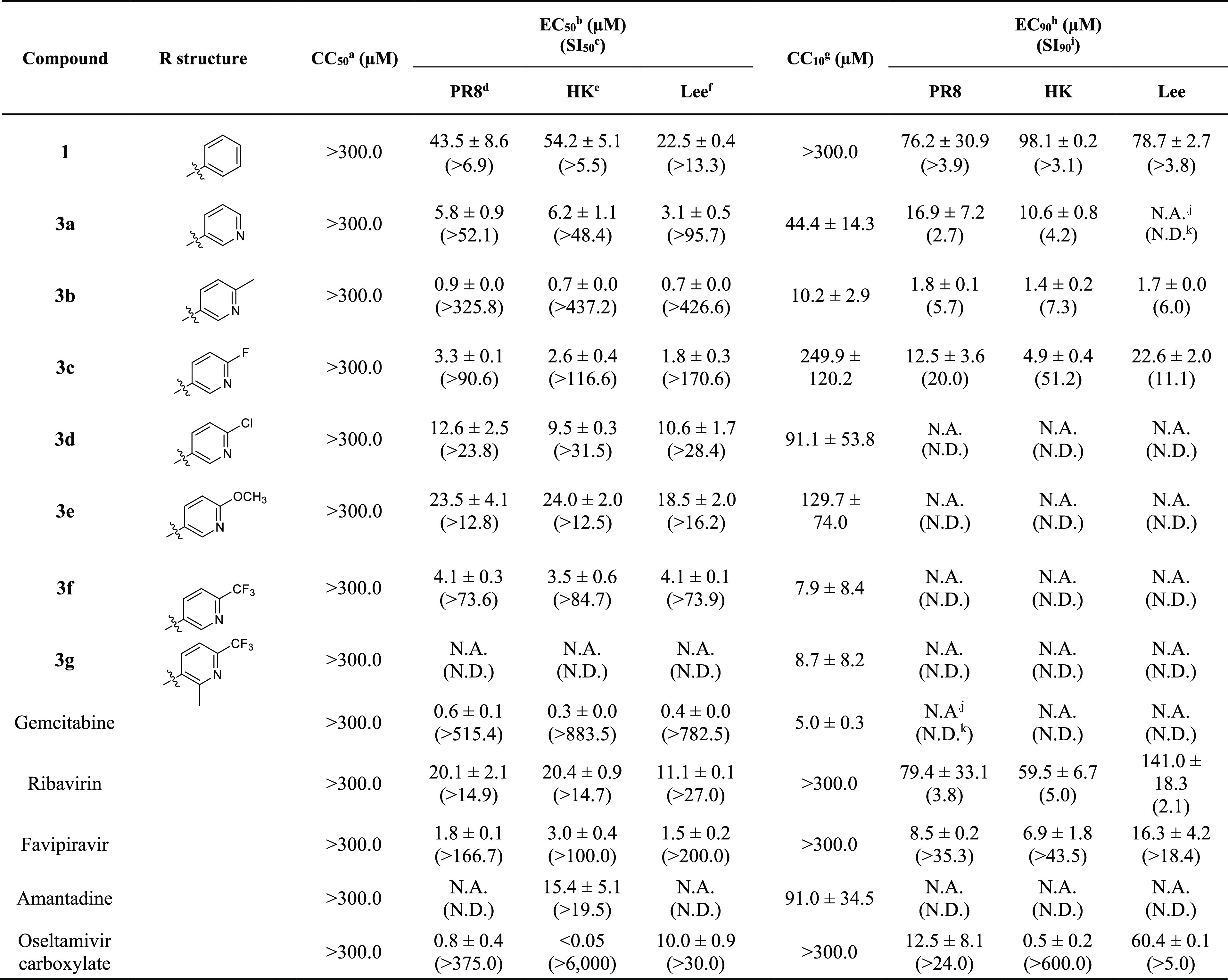
Antiviral Activity of Pyridine-3-carbonyl Substituents

a Compound concentration required for inhibiting cell viability by 50%.

b Compound concentration required for enhancing viability of each virus-infected cells to 50%.

c The ratio of CC_50_ to EC_50_.

d A/Puerto Rico/8/34 (H1N1).

e A/Hong Kong/8/68 (H3N2).

f B/Lee/40.

g Compound concentration required for inhibiting cell viability by 10%.

h Compound concentration required for enhancing viability of each virus-infected cells to 90%.

i The ratio of CC_10_ to EC_90_.

j Not available.

k Not determined.

### Synthesis of Gemcitabine Derivatives and Structure–Activity Relationship Analysis

**Figure 2:**
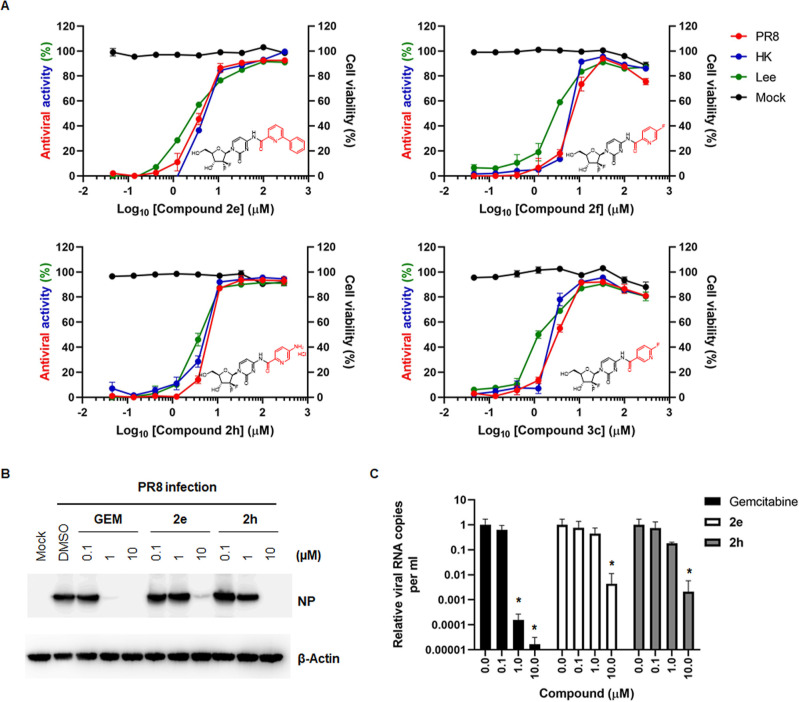
Antiviral activity and cytotoxicity of chemically optimized gemcitabine derivatives, **2e**, **2f**, **2h**, and **3c**. (A) Dose–response curve showing antiviral activity (left *y*-axis) and cytotoxicity (right *y*-axis) of chemically modified compounds from **2a** and **3a**. MDCK cells, mock-infected (black) or infected with three different viruses, PR8 (red), HK (blue), and B/Lee/40 (Lee; green), were treated with increasing concentrations of compounds **2e** (left upper), **2f** (right upper), **2h** (left lower), and **3c** (right lower). On day 3 postinfection, the percentage of viable cells was determined by an MTT assay. Chemical structures are inserted within the graphs, in which modified parts are highlighted in red. Values are expressed as the mean ± SEM from three samples. (B) Inhibition of influenza viral NP expression in the presence of **2e** and **2h**. PR8 virus was used to infect MDCK cells at an MOI of 0.01 for 1 h at 35 °C. The virus-infected cells were treated with 0.1, 1, and 10 μM gemcitabine (GEM), **2e** or **2h**. The following day, cell lysates were harvested for immunoblotting of viral NP by using β-actin as a loading control. The proteins are labeled on the right of the blots. (C) Reduction of viral RNA titers by **2e** and **2h**. Cells were infected and treated with compounds, as shown in (B). Culture supernatants were harvested 1 day postinfection for viral RNA preparation. Two-step quantitative reverse transcription-polymerase chain reaction (qRT-PCR) was performed using an influenza A virus-specific universal primer and an NS gene-specific primer set. Viral genome copies were calculated from Ct value changes relative to those from PR8-infected, DMSO-treated cells. Data are expressed as means ± SEM from three independent experiments. *P*-values below 0.05 were considered statistically significant following a two-way analysis of variance (ANOVA), with Dunnett’s multiple comparison test.

**Scheme 1:**
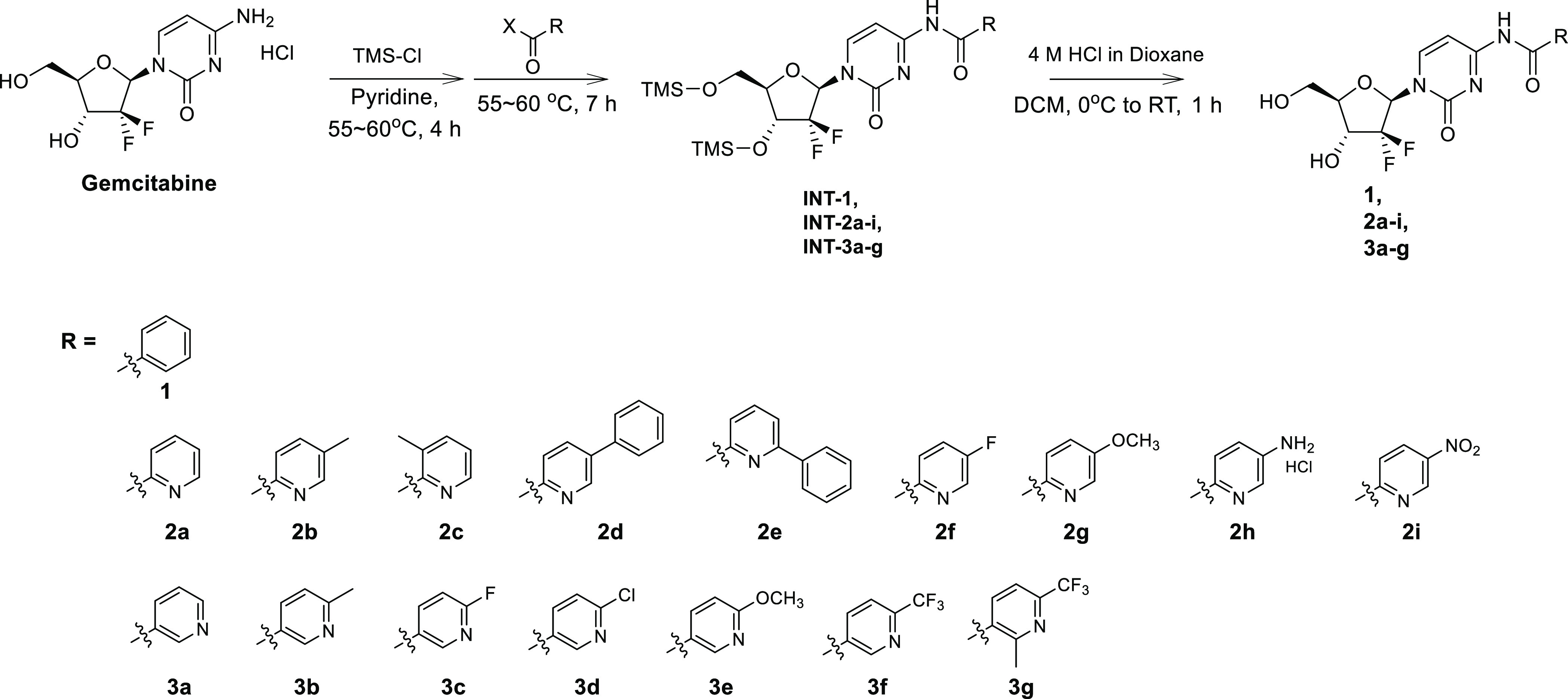
Synthesis of Gemcitabine Derivatives, Compounds **1**, **2a–2i**, and **3a–3g** INT; intermediate. Different substituents (R) are displayed below the synthesis scheme.

Considering antiviral activity with EC_90_ against influenza viruses, because **2a** and **3a** were more potent (EC_90_ values, 1.4–16.9 μM) than compound **1** (EC_90_ values, 76.2–98.1 μM), we speculated that the pyridine ring could be desirable rather than the phenyl ring for structure–activity relationship (SAR) study. Eight **2a** derivatives were synthesized by substituting positions 3, 5, and 6 of the pyridine ring with methyl, phenyl, fluoro, methoxy, amine, or nitro groups, resulting in compounds **2b**–**2i** (Scheme 1 and the Supporting Information). Cell culture-based antiviral assays showed that these compounds are all active (Table 1). Although neither of them showed improved SI_50_ values, they successfully recovered viability of virus-infected cells over 90%. Compounds **2b**, **2c**, **2d**, **2g**, and **2i** inhibited influenza virus infection with SI_90_ values (>1.4) more potently than gemcitabine but less potently than or comparably to **2a**. It was notable that EC_90_ values of compounds **2e**, **2f**, and **2h** (EC_90_, 10.7–34.3 μM) were higher than that of **2a** (EC_90_, 1.4–2.8 μM), but their cytotoxicity was remarkably improved (CC_10_ values: >300 μM for **2e**, **2f**, and **2h** versus 9.9 μM for **2a**), which led to increases in the SI_90_ values (>8.7) against all three influenza viral strains. Dose–response graphs clearly visualized that they are effective at a wider range of concentrations maintaining cell viability over 90% (Figure 2A). The data from SAR analysis suggested that introduction of either a phenyl ring at position 6 or fluorine or amine at position 5 of the terminal pyridine moiety of **2a** is optimal for the enhancement of antiviral selectivity.

Dose–response experiments demonstrated that another hit compound, **3a**, had maximal antiviral efficacy around at a concentration of 33 μM (Figure 1C). This compound showed considerable EC_50_ values ranging from 3.1 to 6.2 μM but did not guarantee a reliable therapeutic window with cell viability over 90%, resulting in variable SI_90_ values between 2.7 and 4.2 limitedly against influenza A viruses (Table 2). We modified the C6 position of its pyridine moiety with the methyl, fluoro, chloro, methoxy, or trifluoromethyl groups, generating compounds **3b**–**3g** (Scheme 1 and the Supporting Information). With EC_50_ values, we observed gradually decreasing efficacy of compounds **3b**, **3c**, **3f**, **3a**, **3d**, and **3e** (Table 2). Interestingly, compound **3g**, which differs from the trifluoromethyl-substituted **3f** only in methylation of 2C, completely lost antiviral activity. Analysis of cell viability with 90% cutoff displayed only compounds **3b** and **3c** to be active. Compound **3b** suppressed influenza viruses with EC_90_ values between 1.4 and 1.8 μM under subtoxic concentrations with a CC_10_ value of 10.2 μM, while compound **3c** had more selective antiviral activity with EC_90_ values between 4.9 and 22.6 μM and a CC_10_ value of 249.9 μM (Table 2 and Figure 2A). SAR analysis informed that C6 position of the pyridine ring of compound **3a** determines antiviral activity as well as cytotoxicity. These data suggested that among **3a** derivatives, **3c** with fluorine at the C6 position is the most optimized antiviral compound.

### Evaluation of Antiviral Activity of the Hit Compounds

From the SAR analysis, compounds **2e**, **2f**, **2h**, and **3c** were discovered to be the promising antiviral agents against influenza A and B virus infections, with the greatest safety profiles. We next evaluated whether they could reduce the level of viral protein or genome. Increasing concentrations (0.1, 1, and 10 μM) of the most potent compounds, **2e** and **2h**, were treated to PR8 virus-infected MDCK cells overnight for these experiments. As gemcitabine used as a control showed severe cytotoxicity at a higher concentration (100 μM), the maximum dose to be treated was restricted at 10 μM (data not shown). Western blot analysis and qRT-PCR clearly demonstrated that both **2e** and **2h** reduced viral NP protein expression and viral RNA copies in a dose-dependent manner (Figure 2B,C). This result ensured that compounds **2e** and **2h**, of which activities were assessed from a colorimetric assay, are not false-positive products but are able to inhibit viral protein expression and progeny virus generation during multiround infection cycles.

### Inhibition of Viral Polymerase Activity by Compounds **2e** and **2h**

**Figure 3:**
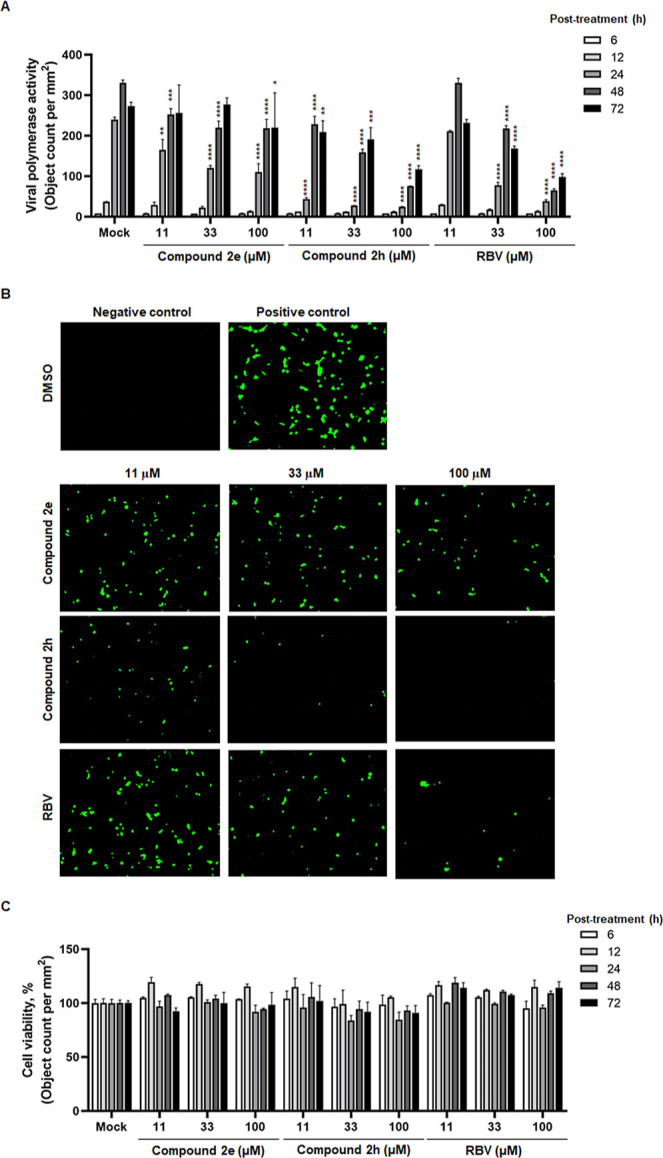
Inhibition of influenza A viral polymerase activity by **2e** and **2h**. (A) Time course dose–response graph of viral polymerase activity in the presence of **2e** and **2h**. HeLa cells were cotransfected with plasmids comprising an influenza viral replicon system amplifying both strands of EGFP transcripts. At 4 h post-transfection, cells were treated with increasing concentrations (11, 33, and 100 μM) of **2e** or **2h** or RBV as a positive control. Polymerase activity was measured by quantifying the number of fluorescent spots per well for 72 h at 6 h intervals. (B) Representative fluorescent microscopy images showing inhibition of EGFP-expressing influenza viral polymerase activity by compounds **2e** and **2h**. Negative control samples were untransfected, while positive control samples were transfected with the minigenome replicon plasmids and 0.2% DMSO-treated. Gemcitabine derivatives, **2e** and **2h**, and RBV were used to treat the transfected HeLa cells at concentrations of 11, 33, and 100 μM. The images were obtained at 24 h post-treatment. Original magnification, ×200. (C) Cytotoxicity of **2e** and **2h**. Samples were prepared as described in (A). Confluence was analyzed by measuring object counts per well on the bright-field microscopy images at different time points. In (A) and (C), values are expressed as means ± SEM from experiments in triplicate. Statistical significance was determined by two-way ANOVA, with Dunnett’s multiple comparison test compared to the mock-treated samples. *, *P* < 0.05; **, *P* < 0.01; ***, *P* < 0.001; ****, *P* < 0.0001.

We examined the effect of compound **2e** or **2h** on the polymerase activity of influenza A virus PR8 in human cells, HeLa. Here, a negative-sense EGFP gene flanked with 5′ and 3′ UTRs derived from the NS segment was cloned under control of the human RNA polymerase I promoter. The viral minigenome construct was subsequently cotransfected with four plasmids expressing viral proteins, PB2, PB1, PA, and NP, under control of the CMV promoter. Likewise vRNA, the negative-sense EGFP RNA is recognized by the viral polymerase complex and NP for the synthesis of its mRNA and positive-sense RNA. Time course analysis of EGFP expression showed that **2h** potently inhibited viral polymerase activity in a dose-dependent manner during 72 h after treatment, when compared to **2e** or RBV (Figure 3A,B). To exclude the possibility that this inhibition was caused by toxicity of the compounds to the transfected cells, viable cells were enumerated from bright-field images of each sample (Figure 3C). There was no significant difference in the cell occupancy between the compound-treated samples and the mock-treated control throughout the entire time course. The polymerase activity analysis confirmed that gemcitabine derivatives, **2e** and **2h**, suppress influenza virus infection by affecting viral RNA replication/transcription in cells.

### In Vivo Anti-Influenza Viral Activity of Compound **2h**

**Figure 4:**
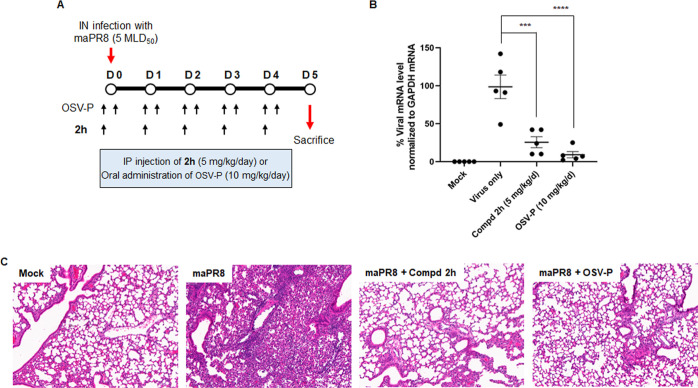
In vivo antiviral activity of **2h** in an influenza virus-infected mouse model. (A) Schematic illustration of in vivo studies using compound **2h** to treat influenza A virus infection. Four hours before intranasal infection with mouse-adapted PR8 strain (maPR8) at a dose of 5 MLD_50_, BALB/c mice were intraperitoneally administered with **2h** (5 mg/kg) or orally with oseltamivir phosphate (OSV-P; 5 mg/kg). At 4 h after virus infection, mice were additionally given the same dose of OSV-P. Mice were subsequently treated daily with **2h** once a day (QD) or with OSV-P twice a day (BID) for additional 4 days. Mock-infected mice and maPR8-infected mice were used as controls. Eight mice were assigned to each group. On day 5, five mice were sacrificed for viral RNA titration in the lungs and the rest three mice for lung histopathology analysis. (B) Reduction of viral RNA copies in the lungs after treatment of mice with **2h**. As described in (A), five mice in each group were sacrificed to prepare total RNA from the lung tissues. Viral mRNA was quantified by qRT-PCR using an oligo(dT) and influenza A virus NS genome-specific primers. GAPDH mRNA level was calculated to normalize the viral mRNA expression. Each symbol represents an individual mouse. Statistical analysis was performed using an ordinary one-way ANOVA, with Dunnett’s multiple comparison test compared to the virus-infected, mock-treated group (virus only). ***, *P* < 0.001; ****, *P* < 0.0001. (C) Histopathological analysis of maPR8-infected lung tissues after treatment with compound **2h**. Three mice in each group were sacrificed at day 5 postinfection, and lung tissues were fixed with formalin for hematoxylin and eosin (H&E) staining. Tissues from mock-infected mice (Mock) and A/H1N1 maPR8 virus-infected mice (maPR8) were used as controls. Images from **2h**-treated mice (maPR8 + Compd 2h) are displayed for comparison with OSV-P-treated samples (maPR8 + OSV-P). Original magnification, ×100.

We next sought to determine whether the chemically optimized gemcitabine derivatives could control influenza A virus infection in an animal model. Our preliminary physicochemical analysis informed that when compounds **2e** and **2h** were dissolved in phosphate-buffered saline (PBS), **2e** appeared aggregated at a concentration of 0.5 mg/mL, whereas **2h** solution was clear at least at 2 mg/mL (data not shown). We therefore selected relatively soluble compound **2h** for in vivo antiviral activity study because the sample had to be prepared in PBS at 2 mg/mL for administration into mice at 5 mg/kg. Mice were intraperitoneally treated with **2h** once daily for 5 days, beginning 4 h prior to mouse-adapted PR8 (maPR8) virus infection. In parallel, oseltamivir phosphate (OSV-P) was orally administered as a control at a dose of 5 mg/kg twice a day, i.e., 10 mg/kg/day, for 5 days (Figure 4A). We investigated whether compound **2h** could alleviate lung damage or reduce viral RNA replication. All mice were sacrificed on day 5 after infection to collect lung samples. From eight mice per group, lung tissues of five mice were dissociated into single cells for total RNA preparation, while tissues of the remaining three animals were fixed with formalin for paraffin embedding for histopathological analysis (Figure 4B,C). qRT-PCR revealed that **2h** inhibited viral mRNA transcription within lung tissues as efficiently as OSV-P (Figure 4B). Lung histopathology analysis demonstrated that treatment with **2h** or OSV-P normalized lung tissues of PR8-infected mice (Figure 4C). In contrast, diffuse alveolar damage and infiltration of inflammatory macrophages or neutrophils were detected in the untreated, virus-infected samples. Taken together, these data suggested that compound **2h** ameliorates lung pathology of influenza virus-infected mice by reducing viral genome replication or mRNA transcription there.

### Inhibition of SARS-CoV-2 Infection by Compound **2h**

**Figure 5:**
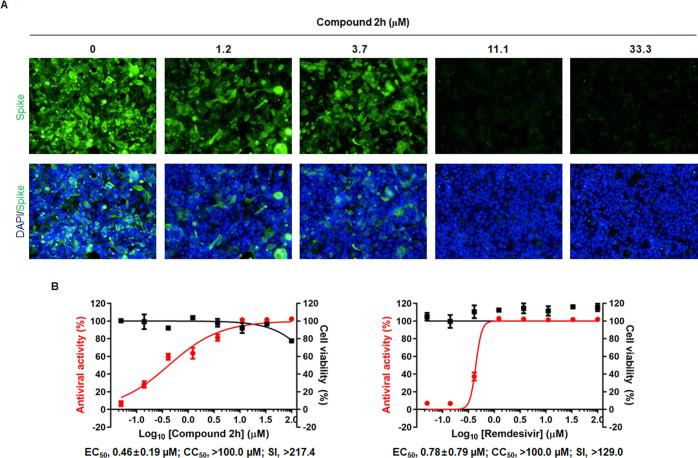
Anti-SARS-CoV-2 activity of compound **2h** in human lung cells. (A) Immunofluorescence microscopy. Calu-3 cells infected with SARS-CoV-2 at an MOI of 0.05 were treated with various concentrations of compound **2h**, 1.2, 3.7, 11.1, and 33.3 μM, for 2 days at 37 °C, where 0.2% DMSO was used as a delivery vehicle control. After fixing and permeabilization, cells were stained with an anti-S antibody and Alexa Fluor 488-conjugated goat anti-mouse IgG (green; upper panels). Cell nuclei were counter stained with DAPI (blue). Merged images are presented in the lower panels. (B) Dose–response curves of antiviral activity and cytotoxicity. SARS-CoV-2-infected Calu-3 cells were treated with increasing concentration of compound **2h** or RDV as depicted in (A). Antiviral activity was determined by normalizing reciprocal fluorescence intensity from SARS-CoV-2-infected cells (red line), while cell viability was estimated by measuring viability of mock-infected cells with MTT (black line). EC_50_, CC_50_, and SI values are recorded below each graph.

We wondered if compound **2h** could also inhibit infection of another respiratory RNA virus, SARS-CoV-2, in human lung cells. As mentioned above, being different from influenza virus with segmented negative-sense RNA genome replicating in the nucleus, SARS-CoV-2 has a nonsegmented positive-sense single-stranded RNA genome replicating in the cytoplasm. To test the broad-spectrum antiviral activity, Calu-3 cells infected with SARS-CoV-2 (multiplicity of infection [MOI], 0.05) were treated with compound **2h** for 2 days by using RDV as a control. Immunofluorescence assay with an anti-S antibody visualized reduction of the viral protein by **2h** in a dose-dependent manner, where nuclei were stained with 4′,6′-diamidino-2-phenylindole (DAPI) comparably in all samples (Figure 5A). Counting of the number of S-positive cells from virus-infected cells together with measurement of cell viability from mock-infected cells indicated that **2h** efficiently inhibited SARS-CoV-2 infection with an EC_50_ value of 0.46 μM and a CC_50_ value above 100 μM, resulting in an SI value of >217.4 (Figure 5B). Even though about 20% cell death was detected in the presence of 100 μM **2h**, its antiviral efficacy or selectivity in human lung cells was comparable to that of RDV (EC_50_, 0.78 μM; CC_50_, >100 μM; and SI, >129.0). The cell culture-based antiviral assay clearly demonstrated that the nucleoside analogue **2h** is active against SARS-CoV-2 as well as influenza virus.

## Discussion

Gemcitabine originally has been approved as an antitumor drug with a mode-of-action blocking DNA replication and mRNA synthesis.^22^ Its treatment causes apoptosis or S-phase cell cycle arrest by terminating DNA or RNA synthesis or by depleting dNTPs through inhibition of ribonucleotide reductase.^21,24^ With evidence of incorporation of gemcitabine triphosphate into viral RNAs, it is comprehensible that this compound has broad-spectrum antiviral activity against RNA viruses, including rhinovirus, rotavirus, enterovirus, influenza virus, and coronavirus.^20,25−30^ As another antiviral machinery, it has been suggested that gemcitabine triggers innate immune response by inhibiting the salvage pathway for pyrimidine synthesis.^27,31^ These multiple functions of gemcitabine render it difficult to simplify that reduction of viral infection is wholly responsible for its direct-acting antiviral efficacy. In other words, as virus cannot actively replicate under a metabolically impaired cytostatic condition, the suppressed viral growth could be confused with antiviral action. Thus, when antiviral activity is assessed by measuring 50% cell viability, it should be carefully proved whether reduction of virus replication arises either from toxicity-mediated poor infectivity or from true inhibitory effect. As an example, Caco-2 human colorectal adenocarcinoma cells are frequently used as susceptible cells for infection of influenza virus or SARS-CoV-2. Irrespective of their availability in antiviral experiments, a recent report suggested that gemcitabine as an antitumor compound suppresses Caco-2 cancer cell proliferation by 40–50% at a wide range of concentrations between 0.1 μM and 1 mM during 3 day incubation.^32^ In a similar manner, our experiments showed that incubation of MDCK cells with gemcitabine gradually decreased cell viability at concentrations above 3.3 μM (Figure 1C). Moreover, in spite of its profound EC_50_ values against influenza viruses (0.3–0.7 μM), antiviral activity failed to recover over 90% cell viability of the virus-infected cells (Table 1). Actually, due to this conflict, cell culture-based antiviral assays frequently are examined by shortening the exposure time less than 24 h or by lowering its dose in a combination treamtent.^20,28,33^ To our knowledge, the present study is a first report to analyze antiviral activity of gemcitabine derivatives against RNA viruses, supporting over 90% cell viability. We propose that SI_50_ values in a SAR study might not be appropriate for selecting druggable antiviral agents, particularly when they possess a cytotoxic core skeleton.

With an aim to reduce gemcitabine’s cytotoxicity, Zheng et al. previously found that a phosphoramidate prodrug of the 4′-fluoro-subsituted gemcitabine analogue (named compound **2b** in the paper) has remarkably improved cytotoxicity profiles and thus more selectively inhibited infection of a DNA virus, varicella zoster virus, compared to gemcitabine.^18^ Unfortunately, it was not able to suppress infection of an RNA virus, such as SARS-CoV-2. Here, we adopted a differentiated chemical modification strategy, in which the amine group of gemcitabine is targeted for conjugation with an aromatic carbonyl ring. Our results showed that the most promising compound, **2h**, diminishes cytotoxicity by over 60-fold (Table 1 and Figure 2). The EC_90_ values of **2h** ranged between 12.2 and 15.9 μM against influenza A and B viruses, but it was not available in gemcitabine. Importantly, efficacy of **2h** was verified in an animal model infected with mouse-adapted influenza A virus (Figure 4). Although in the in vivo study decrease of viral mRNA level and subsequent histopathological improvement were observed after intraperitoneal administration of **2h**, enhancement of mean survival date or dramatic alleviation of body weight decrease was not detected (data not shown). It seems to be responsible for insufficient serum concentration of an active metabolite or too high titer of challenged virus at 5 × 50% mouse lethal dose (MLD_50_).

A pharmacology study elucidated that gemcitabine is metabolized into dFdU by cytidine deaminase in the extracellular as well as cytoplasmic regions, followed by transformation into its mono-, di-, or triphosphorylated metabolites individually in cells.^22^ Given the chemical structure of **2h**, it is assumed that likewise gemcitabine, it can also be metabolized into the three different phosphate forms by intracellular kinases (Figures 1B and 2A). However, being different form gemcitabine, the amine group was chemically modified with an aromatic carbonyl ring, presumably not being easily converted into dFdU (Scheme 1). In another aspect, we cannot exclude the possibility that **2h** produces dFdU via an indirect pathway in which gemcitabine is created after hydrolysis of the amide bond. Regarding metabolism and functionality of **2h**, we have fundamental questions to be addressed. They include whether **2h** is converted into gemcitabine, and if it is, whether the antiviral activity results from the triphosphorylated metabolite of unhydrolized **2h** or of newly created gemcitabine. To address the first question, mice were administered with highly purified **2h** (over 97.3%) for pharmacokinetic study. Preliminary data showed that 17.5% of **2h** is converted to gemcitabine in mice within 30 min (Figure S1 and Table S1). In contrast, the compound was much more stable in a microsomal condition in vitro (Table S2). Collecting the results, **2h** seems to be active in itself but partially metabolized into gemcitabine by a serum component. To understand metabolism and function of **2h** precisely, we are going to synthesize its triphosphate form to test the in vitro inhibitory effect using purified polymerase complex or to quantify serum concentration of the triphosphate form after administration of animals with **2h**.

It is a noteworthy finding that the primarily optimized nucleoside analogue, **2h**, successfully suppresses infection of SARS-CoV-2 as well as influenza viruses with improved cytotoxicity profile (Figure 5). This study provides a framework for chemical modification of gemcitabine to acquire more-active but less-toxic antiviral agents against different RNA viruses. Additional investigation into pharmacokinetics and physiochemical characterization of **2h** for estimating serum stability, bioavailability, and solubility remains for the next-round chemical modifications and preclinical toxicity studies.

## Conclusions

In conclusion, from screening of a nucleos(t)ide analogue-focused chemical library against influenza virus, we found that substitution of the amine group of gemcitabine with an aromatic carbonyl ring is worthy of modification to improve safety. The newly synthesized picolinoyl substituent **2h** inhibited influenza A and B virus infection with EC_90_ values between 11.4 and 15.9 μM but with a CC_10_ value above 300 μM, comparable to favipiravir. The gemcitabine derivative suppressed viral protein expression in cell lysates as well as progeny virus production into culture supernatants in a dose-dependent manner by targeting viral polymerase activity. Decisively, its antiviral activity against influenza virus was evaluated in a mouse model, showing reduced viral mRNA levels as well as normalized lung histopathology at a dose of 5 mg/kg/day. In an independent experiment, it was verified that **2h** was also able to suppress SARS-CoV-2 infection at subtoxic concentrations in human lung cells. This chemical modification strategy provides a plausible approach for the development of another class of cytidine derivatives with improved antiviral selectivity against positive- and negative-sense RNA viruses.

## Methods

### Cells, Viruses, and Chemical Reagents

MDCK, human cervical cancer-derived HeLa, African green monkey kidney Vero, and human lung adenocarcinoma Calu-3 cells were purchased from the American Type Culture Collection (ATCC, Manassas, VA, USA). HeLa and Vero cells were maintained in 10% fetal bovine serum (FBS)-supplemented Dulbecco’s modified Eagle medium (Invitrogen, Carlsbad, CA, USA), while MDCK and Calu-3 cells were cultured in 10%-FBS-supplemented minimum essential medium (MEM; Invitrogen) and Eagle’s MEM (EMEM; Corning, Manassas, VA, USA), respectively.

Influenza A viruses, including PR8 (A/H1N1) and HK (A/H3N2), were obtained from ATCC and amplified in 10- or 11 day-old embryonated chicken eggs. Influenza B virus (Lee strain; ATCC) was inoculated using MDCK cells in serum-free MEM with 2 μg/mL L-1-tosylamido-2-phenyltehyl chloromethyl ketone-treated trypsin (Sigma-Aldrich, St. Louis, MO, USA). SARS-CoV-2 (hCoV-19/Korea/KCDC06/2020) was provided by Korea Disease Control and Prevention Agency (KCDA) and maintained in Vero cells under a serum-free condition. At day 3 postinfection, titers of influenza viruses and SARS-CoV-2 were assessed by plaque assay before storage at −80 °C. All experiments with infectious SARS-CoV-2 were performed within the biosafety level 3 (BSL-3) facility in KRICT.

Gemcitabine (purity, ≥98%), RBV (≥98%), and AMT hydrochloride (≥98%) were purchased from Sigma-Aldrich. Favipiravir (≥98%) and RDV (100%) were acquired from AdooQ BioScience (Irvine, CA, USA). OSV-C (≥98%; United States Biological, Swampscott, MA, USA) was treated for in vitro cell culture-based antiviral assays, while its prodrug, OSV-P (≥98%; Sigma-Aldrich), was orally administered to mice for an in vivo efficacy study.

### Screening of a Nucleos(t)ide Analogue-Focused Chemical Library against Influenza Virus

A small molecule library of 488 nucleos(t)ide analogues (≥95%) composed of known and newly synthesized compounds was provided by ST Pharm Co., Ltd. (Seoul, Republic of Korea). MDCK cells infected with influenza virus PR8 or HK at an MOI of 0.001 were treated with each compound at a final concentration of 10 μM. At day 3 postinfection, reduction of virus-induced CPE was assessed by measuring cell viability after the addition of 2.5 mg/mL of MTT (Sigma-Aldrich).^34^

### Synthesis of Gemcitabine Derivatives

Gemcitabine derivatives were synthesized according to a previously described method.^35^ As shown in the synthesis scheme (Scheme 1), a protection reaction was performed with gemcitabine, followed by an immediate amide reaction with different acyl halides to afford the intermediates (INT-1, INT-**2a–i**, and INT-**3a–g**). Deprotection reactions of the intermediates with 4 M HCl in dioxane solution generated the gemcitabine derivatives **1**, **2a**–**i**, and **3a**–**g** as final products. The structures of these compounds were identified by 300 (Unity Inova; Varian, Palo Alto, CA, USA) or 400 MHz ^1^H NMR spectroscopy (Bruker Avance III 400; Bruker Biospin, Rheinstetten, Germany) as recorded in the Supporting Information and liquid chromatography–mass spectrometry (Agilent 6120 LC/MS System; Agilent Technologies, Santa Clara, CA, USA). Their purity was estimated to be ≥ 97% by high-performance liquid chromatography. For antiviral or cytotoxicity analysis, all compounds were dissolved in DMSO at a final concentration of 50 mM.

### Antiviral and Cytotoxicity Tests

Antiviral activity against influenza virus and cytotoxicity to MDCK cells were assessed by the addition of threefold serial dilutions of each compound, ranging from 300 to 0.05 μM, according to a previous report.^34^ Inhibitory effect on SARS-CoV-2 infection and cytotoxicity to Calu-3 cells were tested by immunofluorescence staining with an anti-S antibody and MTT-based cell viability assay, respectively, according to our previous report with some modifications.^33^ Briefly, Calu-3 cells were seeded on 96-well plates (3 × 10^4^ cells per well). On the next day, cells were treated with threefold serial dilutions of each compound (starting from 100 μM) for 1 h and subsequently mock-infected by the addition of an equal volume of EMEM or infected with SARS-CoV-2 at an MOI of 0.05. On day 2, cell viability was measured by treating mock-infected cells with the MTT solution, while antiviral activity was measured by staining of virus-infected cells with an anti-S antibody, where nuclei were counter-stained with DAPI. The EC_50_ and EC_90_ values were defined as the compound concentrations that increased viability of virus-infected cells to 50 and 90%, respectively. The CC_50_ and CC_10_ values were obtained as compound concentrations that reduced the viability of mock-infected cells by 50 and 10%. These values were derived from three independent experiments using GraphPad Prism 8 software (San Diego, CA, USA). SI_50_ or SI was determined as the ratio of CC_50_ to EC_50_, while SI_90_ was defined as the ratio of CC_10_ to EC_90_. In all experiments, a final concentration of 0.6% (for influenza virus) or 0.2% DMSO (for SARS-CoV-2) was used as a mock control.

### Western Blot Analysis

One day after seeding of MDCK cells in six-well plates (6 × 10^5^ cells per well), they were mock-infected or infected with PR8 virus at an MOI of 0.01 for 1 h at 35 °C. After removal of unadsorbed influenza virus, increasing concentrations of each compound were used to treat cells for an additional day at the same temperature. Cell lysates harvested using M-PER reagent (Thermo Fisher Scientific, Rockford, IL, USA) were subjected to 10% sodium dodecyl sulfate-polyacrylamide gel electrophoresis (SDS-PAGE), followed by electrotransfer to polyvinylidene fluoride membranes (Merck-Millipore, Tullagreen, Ireland). Membranes were probed with antibodies raised against viral NP (Cat. no., 11675-T62; Sino Biological, Beijing, China) and β-actin as a loading control (Cat. no., A1798; Sigma-Aldrich) according to previously described protocols.^34^

### Quantitative RT-PCR

Cell culture supernatants from three independent samples prepared as mentioned above were subject to viral RNA isolation using QIAamp viral RNA mini kit (Qiagen, Hilden, Germany). Complementary DNA was synthesized using SuperScript III reverse transcriptase (Invitrogen, Carlsbad, CA, USA) and the influenza A virus-specific universal primer.^36^ A conserved sequence within the segment 8 NS genome was amplified with primers and a 2× SYBR Green real-time PCR master mix (Toyobo, Osaka, Japan) using a CFX96 real-time PCR detection system (Bio-Rad, Hercules, CA, USA).^37^ Data from compound-treated samples were normalized to virus-infected, untreated samples using Bio-Rad’s CFX Manager Software.

For total RNA purification from lung samples (*n* = 5 per group), mice were sacrificed 5 days postinfection. Lung tissues were homogenized using gentleMACS C Tubes and a gentle MACS Octo Dissociator with Heaters (Miltenyi Biotec, San Diego, CA, USA). Total RNA was prepared using Trizol (Invitrogen) according to the manufacturer’s instructions. After cDNA synthesis with an oligo(dT) primer, viral mRNA was quantified using the NS gene-specific primer by real-time RT-PCR and normalized to the mouse GAPDH mRNA level.^38^

### Cell-Based Viral Polymerase Assay

Polymerase activity of influenza A virus was examined as previously described with the following modifications.^37^ Briefly, HeLa cells seeded in 24-well plates (2 × 10^5^ cells per well) were cotransfected with plasmids pVP-PB2, -PB1, -PA, and -NP expressing PR8 polymerase complex and NP together with a reporter plasmid pHH21-EGFP transcribing a negative-sense EGFP ORF flanked with NS genome-derived 5′- and 3′-UTRs (0.4 μg each). EGFP expression and cell confluency were monitored every 6 h for 72 h using a live cell imaging system (IncuCyte; Essen BioScience, Ann Arbor, MI, USA).

### In Vivo Antiviral Experiments

Six-week-old BALB/c female mice were mock-administered or administered with test compound **2h** intraperitoneally (5 mg/kg, QD) or with OSV-P orally (5 mg/kg, BID) 4 h before intranasal infection with maPR8 at 5 MLD_50_. Four hours later, mice treated with OSV-P were given an additional dose via the same treatment route. maPR8-infected mice continued with the same treatment intervals once (**2h**) or twice (OSV-P) a day for 4 additional days. For analysis of viral RNA and lung histopathology, groups of mice were sacrificed at day 5 postinfection, and lungs were collected postmortem (*n* = 8 per group). For histopathology, tissues (*n* = 3 per group) were fixed with 10% formalin and embedded in paraffin, before preparing 5 μm sections for H&E staining. Bright-field images were captured using a microscope (BX53; Olympus, Waltham, MA, USA) and analyzed using Nuance software v.3.0.2 (Perkin Elmer, Waltham MA, USA). In parallel, total RNA was prepared from dissociated lung cells of the rest mice (*n* = 5 per group) using Trizol reagent (Invitrogen) for qRT-PCR as mentioned above. Animal experiments were conducted in accordance with ethical guidelines approved by the Institutional Animal Care and Use Committee (IACUC) of KRICT with an approval code number of 2021-6D-10-01.

### Statistical Analysis

All experiments were performed in triplicate (for in vitro studies) or in quintuplicate (for in vivo studies). Data are expressed as means ± SEM. Statistical significance between groups was analyzed with an ordinary one-way ANOVA with Dunnett’s multiple comparison tests using the GraphPad Prism software package, version 8.4.3 (GraphPad, San Diego, CA, USA). *P*-values below 0.05 were considered statistically significant.

## Supporting Information Available

Synthesis and characterization of gemcitabine derivatives (PDF)
