# Supplementary material for: Evaluation of Antiviral Activity of Gemcitabine Derivatives against Influenza Virus and Severe Acute Respiratory Syndrome Coronavirus 2
Source: ACS Infect Dis. 2023 Mar 13;9(4):1033–45. doi: 10.1021/acsinfecdis.3c00034 (PMC10081574; doi:10.1021/acsinfecdis.3c00034)
Supplement: Supplementary file 1 — id3c00034_si_001.pdf [file id3c00034_si_001.pdf]

Supporting Information

Evaluation of Antiviral Activity of Gemcitabine Derivatives against Influenza Virus and Severe Acute Respiratory Syndrome Coronavirus 2

Hyeon-Min Cha[†], Uk-Il Kim[†], Soo Bin Ahn, Myoung Kyu Lee, Haemi Lee, Hyungtae Bang, Yejin Jang, Seong Soon Kim, Myung Ae Bae, Kyungjin Kim[*] and Meehyein Kim[*]

[†]These authors contributed equally to this work.

*Corresponding authors

Meehyein Kim, Ph.D.

Infectious Diseases Therapeutic Research Center, Korea Research Institute of Chemical Technology (KRICT), Daejeon 34114, Republic of Korea

Email: mkim@krict.re.kr; Telephone: 82-42-860-7540; Fax: 82-42-860-7400

Kyungjin Kim, Ph.D.

ST Pharm Co., Ltd., Seoul 06194, Republic of Korea

Email: kyungjin.kim@stpharm.co.kr; Telephone: +82-2-527-6351; Fax: +82-2-561-6006

Synthesis and characterization of gemcitabine derivatives

Compound 1

N-(1-((2R,4R,5R)-3,3-difluoro-4-((trimethylsilyl)oxy)-5-(((trimethylsilyl)oxy)methyl)tetrahydrofuran-2-yl)-2-oxo-1,2-dihydropyrimidin-4-yl)benzamide (INT-1): To a solution of gemcitabine (263 mg, 1.00 mmol) in Pyridine (5.0 ml) was added TMSCl (0.28 ml, 2.20 mmol). The mixture was stirred at 55 °C to 60 °C for 3.5 hours, and then benzoyl chloride (0.14 ml, 1.20 mmol) was added and stirred at the same temperature for 7 hours. The reaction solution was diluted with EtOAc and then washed with saturated aqueous $CuSO_4$ solution for 5 times. The organic layer was dried over Na_2SO_4 and concentrated under reduced pressure to afford the **INT-1** (417 mg, 81.5%) as a white solid. ^{1}H-NMR 300 Hz (DMSO-d_6): 11.39 (brs, 1H), 8.22 (d. *J* = 7.5 Hz, 1H), 7.99 (d. *J* = 8.7 Hz, 2H), 7.57 (m. 3H), 7.42 (d. *J* = 7.8 Hz, 1H), 6.24 (t. *J* = 7.5 Hz, 1H), 4.46 (m, 1H), 4.06 (m, 1H), 3.86 (m. 2H), 0.17 (s, 18H); LCMS (ESI, m/z): 512.2 [M+H$^+$].

N-(1-((2R,4R,5R)-3,3-difluoro-4-hydroxy-5-(hydroxymethyl)tetrahydrofuran-2-yl)-2-oxo-1,2-dihydropyrimidin-4-yl)benzamide (1): To a solution of **INT-1** (417 g, 0.815 mmol) in DCM (1.0 ml) was slowly added 4M HCl in dioxane (2 ml) at 0 °C. After stirring at room temperature for 1 hour, the reaction solution was concentrated under reduced pressure. The residue was diluted with purified water, and 2N NaOH aqueous solution was added to adjust the pH to 6~7. The precipitated solid was filtered and dried to afford **1** (152.0 mg, 50.8%) as a white solid. ^{1}H-NMR 300 Hz (DMSO-d_6): 11.37 (brs, 1H), 8.29 (d. *J* = 7.2 Hz, 1H), 7.98 (d. *J* = 8.7 Hz, 2H), 7.57 (m. 3H), 7.37 (d. *J* = 7.5 Hz, 1H), 6.19 (t. *J* = 7.5 Hz, 1H), 4.20 (m, 1H), 3.90 (m, 1H), 3.75 (m. 2H); LCMS (ESI, m/z): 368. [M+H$^+$].

Compound 2a

N-(1-((2R,4R,5R)-3,3-difluoro-4-hydroxy-5-(hydroxymethyl)tetrahydrofuran-2-yl)-2-oxo-1,2-dihydropyrimidin-4-yl)picolinamide (2a): 2a was synthesized in the same manner as described in compound **1** using picolinoyl chloride hydrochloride. ^{1}H-NMR 400 Hz (DMSO-d_6): 9.81 (s, 1H), 8.72 (s, 1H), 8.68 (s, 1H), 8.13 (d, *J* = 8.0 Hz, 1H), 8.04 (m, 2H), 7.67 (m, 1H), 6.18 (d, *J* = 8.0 Hz, 1H), 6.08 (m, 1H), 4.18 (m, 1H), 3.91 (m, 1H), 3.79 (m, 2H); LCMS (ESI, m/z): 368.2 [M+H$^+$].

Compound 3a

N-(1-((2R,4R,5R)-3,3-difluoro-4-hydroxy-5-(hydroxymethyl)tetrahydrofuran-2-yl)-2-oxo-1,2-

dihydropyrimidin-4-yl)nicotinamide (3a): 3a was synthesized in the same manner as described in compound **1** using nicotinoyl chloride hydrochloride. ^{1}H-NMR 300 Hz (DMSO-d_6): 11.61 (s, 1H), 9.09 (s, 1H), 8.76 (d, J = 4.8 Hz, 1H), 8.32 (m, 2H), 7.54 (dd, J = 7.5 Hz, 4.8 Hz, 1H), 7.39 (d, J = 7.8 Hz, 1H), 6.34 (d, J = 6.6 Hz, 1H), 6.20 (t, J = 7.2 Hz, 1H), 5.33 (brs, 1H), 4.19 (m, 1H), 3.91 (m, 1H), 3.74 (m, 2H); LCMS (ESI, m/z): 369.0 [M+H$^+$].

Compound 2b

N-(1-((2R,4R,5R)-3,3-difluoro-4-hydroxy-5-(hydroxymethyl)tetrahydrofuran-2-yl)-2-oxo-1,2-dihydropyrimidin-4-yl)-5-methylpicolinamide (2b): ^{1}H-NMR 400 Hz (DMSO-d_6): 10.53 (s, 1H), 8.61 (m, 1H), 8.40 (d, J = 7.5 Hz, 1H), 8.10 (d, J = 8.0 Hz, 1H), 7.94 (dd, J = 8.0 Hz, 1.32 Hz, 1H), 7.46 (d, J = 7.5 Hz, 1H), 6.34 (d, J = 6.5 Hz, 1H), 6.21 (t, J = 7.2 Hz, 1H), 5.34 (t, J = 4.8 Hz, 1H), 4.20 (m, 1H), 3.91 (m, 1H), 3.82 (m, 1H), 3.67 (m, 1H), 2.44 (s, 3H); LCMS (ESI, m/z): 383.0 [M+H$^+$].

Compound 2c

N-(1-((2R,4R,5R)-3,3-difluoro-4-hydroxy-5-(hydroxymethyl)tetrahydrofuran-2-yl)-2-oxo-1,2-dihydropyrimidin-4-yl)-3-methylpicolinamide (2c): ^{1}H-NMR 400 Hz (DMSO-d_6): 10.89 (s, 1H), 8.56 (m, 1H), 8.37 (d, J = 7.5 Hz, 1H), 7.88 (d, J = 7.2 Hz, 1H), 7.62 (dd, J = 7.8 Hz, 4.6 Hz, 1H), 7.44 (d, J = 7.5 Hz, 1H), 6.34 (d, J = 6.5 Hz, 1H), 6.21 (t, J = 7.3 Hz, 1H), 5.34 (t, J = 5.4 Hz, 1H), 4.21 (m, 1H), 3.91 (m, 1H), 3.82 (m, 1H), 3.67 (m, 1H), 2.63 (s, 3H); LCMS (ESI, m/z): 383.0 [M+H$^+$].

Compound 2d

N-(1-((2R,4R,5R)-3,3-difluoro-4-hydroxy-5-(hydroxymethyl)tetrahydrofuran-2-yl)-2-oxo-1,2-dihydropyrimidin-4-yl)-5-phenylpicolinamide (2d): ^{1}H-NMR 400 Hz (DMSO-d_6): 10.58 (s, 1H), 9.11 (d, J = 1.6 Hz, 1H), 8.43 (m, 2H), 8.27 (d, J = 8.8 Hz, 1H), 7.88 (m, 2H), 7.55 (m, 4H), 6.33 (d, J = 6.4 Hz, 1H), 6.22 (t, J = 7.2 Hz, 1H), 5.33 (t, J = 5.6 Hz, 1H), 4.22 (m, 1H), 3.93 (m, 1H), 3.86 (m, 1H), 3.69 (m, 1H); LCMS (ESI, m/z): 445.0 [M+H$^+$].

Compound 2e

N-(1-((2R,4R,5R)-3,3-difluoro-4-hydroxy-5-(hydroxymethyl)tetrahydrofuran-2-yl)-2-oxo-1,2-dihydropyrimidin-4-yl)-6-phenylpicolinamide (2e): ^{1}H-NMR 400 Hz (DMSO-d_6): 10.62 (brs, 1H), 8.43 (d, J = 7.2 Hz, 1H), 8.32 (d, J = 7.6 Hz, 1H), 8.21 (m, 4H), 7.56 (m, 4H), 6.35 (d, J = 6.4 Hz,

1H), 6.23 (t, J = 6.8 Hz, 1H), 5.35 (t, J = 5.2 Hz, 1H), 4.23 (m, 1H), 3.92 (m, 1H), 3.84 (m, 1H), 3.68 (m, 1H); LCMS (ESI, m/z): 445.0 [$M+H^+$].

Compound 2f

N-(1-((2R,4R,5R)-3,3-difluoro-4-hydroxy-5-(hydroxymethyl)tetrahydrofuran-2-yl)-2-oxo-1,2-dihydropyrimidin-4-yl)-5-fluoropicolinamide (2f): ^{1}H-NMR 400 Hz (DMSO-d_6): 10.49 (s, 1H), 8.79 (d, J = 2.8 Hz, 1H), 8.40 (d, J = 7.6 Hz, 1H), 8.28 (dd, J = 8.7 Hz, 4.5 Hz, 1H), 8.04 (m, 1H), 7.44 (d, J = 7.5 Hz, 1H), 6.34 (d, J = 6.5 Hz, 1H), 6.21 (t, J = 7.2 Hz, 1H), 5.34 (t, J = 5.5 Hz, 1H), 4.21 (m, 1H), 3.92 (m, 1H), 3.82 (m, 1H), 3.67 (m, 1H); LCMS (ESI, m/z): 387.0 [$M+H^+$].

Compound 2g

N-(1-((2R,4R,5R)-3,3-difluoro-4-hydroxy-5-(hydroxymethyl)tetrahydrofuran-2-yl)-2-oxo-1,2-dihydropyrimidin-4-yl)-5-methoxypicolinamide (2g): ^{1}H-NMR 400 Hz (DMSO-d_6): 10.40 (s, 1H), 8.44 (d, J = 2.4 Hz, 1H), 8.38 (d, J = 7.2 Hz, 1H), 8.18 (d, J = 8.8 Hz, 1H) 7.65 (dd, J = 8.8 Hz, 2.4 Hz, 1H), 7.45 (d, J = 7.6 Hz, 1H), 6.35 (d, J = 4.4 Hz, 1H), 6.21 (t, J = 7.2 Hz, 1H), 5.34 (t, J = 5.2 Hz, 1H), 4.21 (m, 1H), 3.94 (m, 4H), 3.83 (m, 1H), 3.67 (m, 1H); LCMS (ESI, m/z): 399.0 [$M+H^+$].

Compound 2h

5-amino-N-(1-((2R,4R,5R)-3,3-difluoro-4-hydroxy-5-(hydroxymethyl)tetrahydrofuran-2-yl)-2-oxo-1,2-dihydropyrimidin-4-yl)picolinamide.HCl (2h): ^{1}H-NMR 400 Hz (DMSO-d_6): 10.48 (brs, 1H), 8.35 (d, J = 7.5 Hz, 1H), 8.01 (d, J = 2.4 Hz, 1H), 7.90 (d, J = 8.6 Hz, 1H) 7.46 (d, J = 7.6 Hz, 1H), 7.08 (dd, J = 8.6 Hz, 2.6 Hz, 1H), 6.19 (t, J = 7.4 Hz, 1H), 4.20 (m, 1H), 3.91 (m, 1H), 3.82 (m, 1H), 3.66 (m, 1H); LCMS (ESI, m/z): 384.0 [$M+H^+$](free base).

Compound 2i

N-(1-((2R,4R,5R)-3,3-difluoro-4-hydroxy-5-(hydroxymethyl)tetrahydrofuran-2-yl)-2-oxo-1,2-dihydropyrimidin-4-yl)-5-nitropicolinamide (2i): ^{1}H-NMR 400 Hz (DMSO-d_6): 10.72 (s, 1H), 9.49 (dd, J = 2.5 Hz, 0.5 Hz, 1H), 8.84 (dd, J = 8.6 Hz, 2.5 Hz, 1H), 8.41 (m, 2H), 7.43 (d, J = 7.5 Hz, 1H), 6.35 (d, J = 6.5 Hz, 1H), 6.22 (t, J = 8.0 Hz, 1H), 5.35 (t, J = 5.5 Hz, 1H), 4.22 (m, 1H), 3.93 (m, 1H), 3.82 (m, 1H), 3.67 (m, 1H); LCMS (ESI, m/z): 414.0 [$M+H^+$].

Compound 3b

N-(1-((2R,4R,5R)-3,3-difluoro-4-hydroxy-5-(hydroxymethyl)tetrahydrofuran-2-yl)-2-oxo-1,2-dihydropyrimidin-4-yl)-6-methylnicotinamide (3b): ^{1}H-NMR 400 Hz (DMSO-d_6): 9.88 (s, 1H), 9.01 (d, *J* = 1.8 Hz, 1H), 8.76 (s, 1H), 8.39 (dd, *J* = 8.2 Hz, 2.0 Hz, 1H), 8.14 (d, *J* = 7.9 Hz, 1H), 7.62 (d, *J* = 9.0 Hz, 1H), 6.20 (d, *J* = 7.9 Hz, 1H), 6.08 (m, 1H), 4.18 (m, 1H), 3.91 (m, 1H), 3.79 (m, 1H), 3.64 (m, 1H), 2.64 (s, 3H); LCMS (ESI, m/z): 383.1 [M+H$^+$].

Compound 3c

N-(1-((2R,4R,5R)-3,3-difluoro-4-hydroxy-5-(hydroxymethyl)tetrahydrofuran-2-yl)-2-oxo-1,2-dihydropyrimidin-4-yl)-6-fluoronicotinamide (3c): ^{1}H-NMR 400 Hz (DMSO-d_6): 11.67 (s, 1H), 8.91 (dd, *J* = 44.9 Hz, 2.1 Hz, 1H), 8.54-8.35 (m, 2H), 7.70-7.34 (m, 2H), 6.34 (d, *J* = 6.5 Hz, 1H), 6.21 (t, *J* = 6.8 Hz, 1H), 5.33 (t, *J* = 5.4 Hz, 1H), 4.21 (m, 1H), 3.91 (m, 1H), 3.82 (m, 1H), 3.66 (m, 1H); LCMS (ESI, m/z): 387.0 [M+H$^+$].

Compound 3d

6-chloro-N-(1-((2R,4R,5R)-3,3-difluoro-4-hydroxy-5-(hydroxymethyl)tetrahydrofuran-2-yl)-2-oxo-1,2-dihydropyrimidin-4-yl)nicotinamide (3d): ^{1}H-NMR 400 Hz (DMSO-d_6): 11.70 (s, 1H), 8.95 (d, *J* = 2.2 Hz, 1H), 8.35 (m, 2H), 7.69 (d, *J* = 8.4 Hz, 1H), 7.37 (m, 1H), 6.34 (d, *J* = 6.5 Hz, 1H), 6.21 (t, *J* = 7.2 Hz, 1H), 5.34 (t, *J* = 5.4 Hz, 1H), 4.21 (m, 1H), 3.91 (m, 1H), 3.82 (m, 1H), 3.66 (m, 1H); LCMS (ESI, m/z): 403.0 [M+H$^+$].

Compound 3e

N-(1-((2R,4R,5R)-3,3-difluoro-4-hydroxy-5-(hydroxymethyl)tetrahydrofuran-2-yl)-2-oxo-1,2-dihydropyrimidin-4-yl)-6-methoxynicotinamide (3e): ^{1}H-NMR 400 Hz (DMSO-d_6): 11.45 (s, 1H), 8.84 (d, *J* = 2.0 Hz, 1H), 8.31 (m, 1H), 8.25 (dd, *J* = 8.8 Hz, 2.5 Hz, 1H), 7.38 (d, *J* = 6.8 Hz, 1H), 6.93 (d, *J* = 8.7 Hz, 1H), 6.34 (d, *J* = 6.5 Hz, 1H), 6.21 (t, *J* = 7.2 Hz, 1H), 5.33 (t, *J* = 5.4 Hz, 1H), 4.21 (m, 1H), 3.91 (m, 4H), 3.82 (m, 1H), 3.66 (m, 1H); LCMS (ESI, m/z): 399.1 [M+H$^+$].

Compound 3f

N-(1-((2R,4R,5R)-3,3-difluoro-4-hydroxy-5-(hydroxymethyl)tetrahydrofuran-2-yl)-2-oxo-1,2-dihydropyrimidin-4-yl)-6-(trifluoromethyl)nicotinamide (3f): ^{1}H-NMR 400 Hz (DMSO-d_6): 11.80 (s, 1H), 9.24 (s, 1H), 8.58 (dd, *J* = 8.4 Hz, 2.0 Hz, 1H), 8.35 (m, 1H), 8.08 (d, *J* = 8.4 Hz, 1H), 7.38 (m, 1H), 6.32 (d, *J* = 6.4 Hz, 1H), 6.21 (t, *J* = 7.2 Hz, 1H), 5.31 (t, *J* = 5.2 Hz, 1H), 4.21 (m, 1H), 3.91 (m, 1H), 3.82 (m, 1H), 3.67 (m, 1H); LCMS (ESI, m/z): 437.1 [M+H^{+}].

Compound 3g

N-(1-((2R,4R,5R)-3,3-difluoro-4-hydroxy-5-(hydroxymethyl)tetrahydrofuran-2-yl)-2-oxo-1,2-dihydropyrimidin-4-yl)-2-methyl-6-(trifluoromethyl)nicotinamide (3g): ^{1}H-NMR 400 Hz (DMSO-d_6): 11.67 (s, 1H), 8.37 (d, *J* = 7.6 Hz, 1H), 8.20 (d, *J* = 7.9 Hz, 1H), 7.86 (d, *J* = 8.0 Hz, 1H), 7.38 (d, *J* = 7.5 Hz, 1H), 6.34 (d, *J* = 6.5 Hz, 1H), 6.20 (t, *J* = 7.2 Hz, 1H), 5.33 (t, *J* = 5.4 Hz, 1H), 4.22 (m, 1H), 3.91 (m, 1H), 3.82 (m, 1H), 3.67 (m, 1H), 2.62 (s, 3H); LCMS (ESI, m/z): 451.0 [M+H^{+}].

Supplementary Figure and Tables

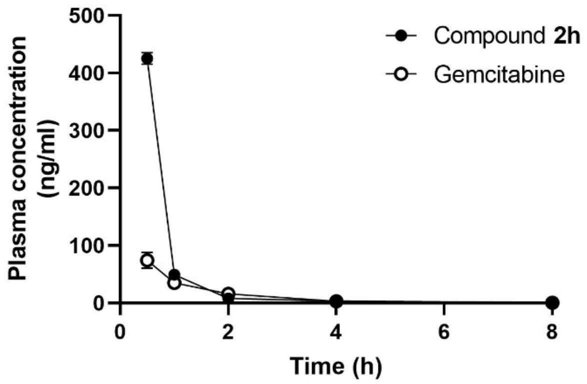

Supplementary Figure S1. Pharmacokinetics of compound 2h in mice. Male BALB/c mice (8-week-old; n = 3) were intraperitoneally treated with compound **2h** (purity, 97.32%) at a dose of 5 mg/kg. At 0.5, 1, 2, 4 and 8 h after treatment, serum samples were collected for measuring plasma concentrations of compound **2h** (filled circle) and metabolized gemcitabine (empty circle).

Supplementary Table S1. Pharmacokinetic parameters of compound 2h in male BALB/c mice.

Parameter (unit)	Compound 2h	Gemcitabine
T_{max} (h)	0.50 ± 0.00	0.50 ± 0.00
C_{max} (μg/mL)	0.43 ± 0.01	0.07 ± 0.01
$T_{1/2}$ (h)	2.09 ± 0.55	1.08 ± 0.30
AUC_t (μg·h/mL)	0.28 ± 0.28	0.10 ± 0.00

T_{max}, time for C_{max}
C_{max}, maximum plasma concentration
$T_{1/2}$, terminal half-life
AUC_t, areas under the plasma concentration-time curve

Supplementary Table S2. Liver microsomal stability (%) at 30 min

Test compound	Mouse	Human
Gemcitabine	90.1 ± 9.7	94.2 ± 8.5
**2h**	>99.9	>99.9
Buspirone	0.3 ± 0.1	14.8 ± 1.8
